# Rat Bait, Not Healthy Rice!

**DOI:** 10.3390/toxics11010060

**Published:** 2023-01-08

**Authors:** Kuan-I Lee, Jing-Hua Lin, Yen-Jung Chu, Jou-Fang Deng, Wei-Lan Chu, Dong-Zong Hung

**Affiliations:** 1 Department of Emergency, Buddhist Tzu Chi General Hospital, Taichung Branch, Taichung 42721, Taiwan; 2 Division of Toxicology, China Medical University Hospital, Taichung 40447, Taiwan; 3 Department of Emergency Medicine, China Medical University Hospital, Taichung 40447, Taiwan; 4 Division of Clinical Toxicology & Occupational Medicine, Department of Medicine, Taipei Veterans General Hospital, Taipei 11217, Taiwan

**Keywords:** bromadiolone, rat bait, vitamin K1

## Abstract

Bromadiolone, a potent, long-acting anticoagulant rodenticide is frequently tinted to a red or pink color and mixed with cereals as rat bait. Six peoples working in a small factory suffered from a severe bleeding tendency several weeks after consuming a rice meal that was tainted with bromadiolone mistaken to be healthy food. High serum levels of bromadiolone and excessive bleeding were found in these individuals, and they needed vitamin K1 therapy for weeks. These cases indicated that long-acting anticoagulant rodenticide might induce cumulative toxicity in repeated, low-dose exposure, and the blood levels of bromadiolone might be an indicator for antidote therapy if available.

## 1. Introduction

Bromadiolone, a second-generation, long-acting anticoagulant rodenticide (LAAR) with a white to yellow color chemically, is marketed worldwide and frequently colored with red dye and mixed with grains as rat bait of 0.005% *w/w* in general meaning that it might be confused with healthy food such as red yeast rice. Long-acting anticoagulant rodenticides have an extremely higher affinity to vitamin K epoxide reductase (VKOR), a key enzyme in the liver for vitamin K reactivation, compared with warfarin. The tight binding and inhibition of VKOR by LAAR allows for a significant deficiency in the reduced form of vitamin K and this is characterized by prolonged coagulopathy and bleeding after initial treatment and the need for high-dose vitamin K1 therapy after LAAR poisoning [1]. The half-life of bromadiolone in human beings has been reported to be as long as 144 h [2]. Repeated small-dose exposures had been suggested to be likely to produce cumulative toxicity and severe coagulopathy [1], but it has never been reported in the literature before. Here, we describe six individuals suffering from varied doses of bromadiolone through repeated exposure with severe bleeding diathesis due to the misuse of rat-bait rice as healthy food for weeks.

## 2. Case Report

**Figure 1:**
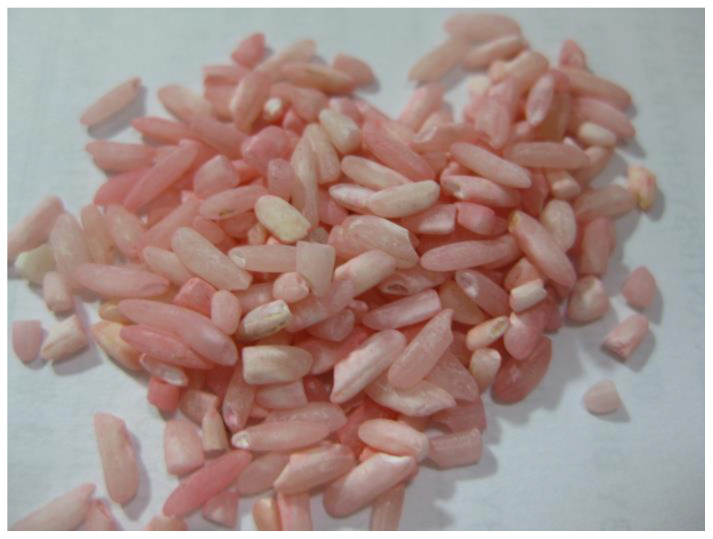
The appearance of the bait. The rice grains were stained unevenly by pink bromadiolone powder before being washed (the patient provided the rice grains).

Six individuals in Taiwan, working overseas from a small company in China, presented to an emergency department (ED) due to hematemesis, hematuria, and multiple ecchymoses on their skin. Tracing back their histories, the first case (patient 1) sought medical attention in a local hospital in China because of severe hematuria, gum bleeding, tarry feces, and ecchymoses for 1–2 days. An unexplained bleeding tendency with a prolonged prothrombin time (PT) was found there. His condition improved and he was released from hospital after a transfusion of fresh frozen plasma. Unfortunately, the hemorrhagic symptoms reappeared and his colleagues subsequently experienced similar symptoms. All of them went back to the emergency room under the suspicion of massive food poisoning due to LAAR-contaminated rice (Figure 1), which their cook mistook as red yeast rice and it was served three times a day. The tainted rice containing 0.5% *w/w* of bromadiolone was prepared to be 0.005% *w/w* of bait. These patients consumed 1–3 bowls of rice per meal and they had lasted for more than 2 weeks before bleeding diathesis occurred. Without rice washing, a bowl of rice is estimated to contain 9 milligrams of rat poison. 

These people all denied any trauma or history of anticoagulants use. Most of them presented with hematuria, gum bleeding, and subcutaneous ecchymosis (5/6 patients). All of them were free from any signs or symptoms of central nervous system damage. Their clinical manifestations are summarized in Table 1. All of them were admitted and received intravenous vitamin K1 therapy until their INR values were normal. Twenty to thirty mg of vitamin K1 intravenously per day in divided doses was prescribed and was adjusted with the INR values being checked every 2–3 days. These patients were discharged and followed-up at an out-patient clinic while their antidotes were shifted to an oral route if they could keep their normal INR value under only 10 mg of vitamin K1 injection per day. All of them recovered with normal INR values and they were free of symptoms after vitamin K1 therapy when they were discharged from hospital. Their blood samples were taken when they were admitted and quantitated by LC-MS/MS in the Toxicology Laboratory of Taipei Veterans General Hospital [3], and high serum bromadiolone (3.9~74.5 ng/mL) levels were identified in all of these six patients.

## 3. Discussion

Bromadiolone, a kind of 4-hydroxycoumarin, is a popular LAAR [1]. Compared with the 17-h half-life of warfarin, the elimination half-life of bromadiolone in animal blood is much longer, about 1.0 to 2.4 days, and even up to 170–318 days in rat livers [1,4]. In case reports of human poisoning [2,5], bromadiolone was eliminated from the blood with the terminal half-life of 10–24 days, contrary to the first phase of 3.5–6 days. The fact of slow elimination suggests the possibility of the cumulative toxicity of bromadiolone in cases of long-term, repeated exposure, similar to the behavior of heavy metal lead or cadmium [1].

Bromadiolone is commonly prepared as a 0.5% red to pink color liquid formulation to be mixed with grains such as rice and corn to a 0.005% *w/w* concentration and it is commonly applied in buildings as rat bait in China [6]. Due to the colorful appearance and the lack of warning signs, it could easily be served as a meal by mistake. Rice is usually washed with water before cooking, and the concentration of leftover bromadiolone in a rice meal might be less than 0.005%. Bromadiolone is thermally stable below 200 degrees Celsius [7]. In cases without washing, a bowl of rice is calculated to contain 9 mg of rat poison. Although we did not obtain the sample for a toxin assay, it was clear from these patients’ data that more serious exposure through continuous ingestion rather than intermittent exposure resulted in higher blood levels, more rapid onset, and severe manifestations. Patient 1, who consumed meals three times a day and 2–3 bowls/meal, suffered from prompt bleeding diathesis and his blood rodenticide level was found to be more than 74.5 ug/L; patient 5 with intermittent exposure and the latest presentation had the lowest blood level and required low doses of vitamin K1 therapy and subsequently had the shortest period of treatment. This suggested that long-term and repeated exposure to such small doses of bromadiolone might have also induced cumulative toxicity and led to a bleeding tendency in humans. According to these small groups of patients, the most frequent presentations were hematuria and gum bleeding with varied serum concentrations of LAAR.

Long term vitamin K1 therapy to prevent a relapse in bleeding tendency and the absence of reliable clinical indicators have been noted in acute LAAR poisoning [1,8]. A few reports from cases studies have indicated that serum bromadiolone levels less than 10 ng /mL and 4–10 ng/mL of brodifacoum are associated with a consistently normal coagulation profile without antidote therapy [1,2,5]. These findings seemed to not be applicable in these cases of low-dose and repeated LAAR exposure. The patients who suffered from significant bleeding and abnormal INR values required the use of vitamin K1 therapy even with serum bromadiolone level less than 4 ng/mL [7]. 

Our study has some limitations. Firstly, these patients could not correctly recall how much contaminated rice they had consumed. Secondly, we did not obtain the rice sample for a bromadiolone assay due to restrictions on the importation of agricultural products. Thirdly, we did not study the toxicokinetics of bromadiolone in these patients to define the antidotes use. However, we confirmed their bromadiolone exposure by detecting significant toxin bromadiolone levels by the LC-MS/MS in the patients’ blood, and its possible relationship to exposure frequency.

In conclusion, low-dose and repeated exposure to LAARs could lead to significant coagulopathy and bleeding from the vital organs and there is a need for prompt antidote therapy regardless of the LAAR blood level.

## Figures and Tables

**Table 1:** Clinical and demographic data of the six patients with bromadiolone intoxication.

	Patient 1 *	Patient 2	Patient 3	Patient 4	Patient 5	Patient 6
Age	58	32	54	32	29	27
Sex	M	M	M	M	F	M
Hematuria	+	+	+	-	+	+
Gum bleeding	+	+	−	+	+	+
GI bleeding	+	−	−	+	−	−
Ecchymosis	+	+	+	+	−	+
Conjunctival hemorrhage	−	−	+	−	−	−
PT/INR (Normal 0.8–1.2)	Unclotted	Unclotted	Unclotted	17.5/1.55	37.7/3.31	25.1/2.21
SBL	74.5	6.17	8.94	7.5	3.9 #	8.0
Total dose of Vit. K1 ^&^ (mg), IV/Oral	130/50	120/100	110/50	120/100	50/0	40/0

*: The patient consumed meals every day during that time and was lost to the follow-up after discharge. SBL: serum bromadiolone level, ng/mL, assayed by LC-MS/MS; #: The patient delayed medical attention and blood tests for three days. ^&^: IV form 10 mg/vial and oral form 5 mg/tablet. Patient 1 was the manager for machine maintenance, patients 2 and 4 were the assistants of the manager, patient 3 was the boss, and patients 5 and 6 were the daughter of the boss and her husband, respectively. The patients 5 and 6 frequently went out on business and might have been less exposed.

## Data Availability

Not applicable.
